# Semen parameters of non-infertile smoker and non-smoker men

**Published:** 2012-12-25

**Authors:** R Davar, L Sekhavat, N Naserzadeh

**Affiliations:** * Research and Clinical Center for Infertility, Shahid Sadoughi University of Medical Sciences, Yazd, Iran; ** Ali Ebn Abitaleb Medical Faculty, Islamic Azad University, Yazd, Iran; *** Ali Ebn Abitaleb Medical Faculty, Islamic Azad University, Yazd, Iran

**Keywords:** semen, smokers, non-smokers, cigarette, non-infertile

## Abstract

**Introduction:** According to the world Health organization, approximately one third of the world` population older than 15 years are smokers. Evidences indicate that both in men and in women, cigarette smoking affects reproductive health more than the consumption of caffeine or alcohol in comparable doses. The mechanisms according to which tobacco affects spermatozoa are poorly understood. Some of the studies focused on the relation between cigarette smoking and the principal semen analysis, variable such as concentration, Morphology and Motility. In this study, we compared the sperm parameters between smokers and non-smokers.

** Material and Methods:** This case control study was done on men from infertile couples who were referred to Yazd research and clinical center for infertility but the cause of infertility was not the male factor. The semen analysis was compared between the smokers and non-smokers.

** Results: **151 infertile men were enrolled in the present study. 98 non-smokers and 53 smokers the mean age of patients was not significantly different between groups. There were no significant differences among groups according to sperm concentration (P-Value 0.108), Morphology (P-Value 0.138) and Motility (P-Value 0.082). Also there were no significant relationship between sperm parameters and the amount of cigarettes people had used (based on Pack/year scale).

**Conclusion:** Semen parameters (Morphology, Motility and concentration) were lower in smokers but there were no significant differences between groups.

## Introduction

Infertility is defined as the inability of a couple to conceive after 12 months of regular, unprotected intercourse. The rate of infertility is about 10%-15% worldwide [**1**] and about 50-80 million couple suffer from this problem that based on new studies smoking is one of the risk factors of male infertility. WHO has estimated that 1/3 of people over 15 years and totally 1.3 billon people in the world are smokers [**2**]. Based on latest information, 47% of men and 12% of women in the world are smokers. In Iran this rates are 25% and 2.5% for men and women respectively [**3**]. Smoking has proofed effects on about 25 diseases like cardiovascular diseases, lung cancer, bronchitis, urinary tract malignancies and several others. Smoking can also have impact on fertility of men and women, even wives of smoker men as passive smokers can affect from smoking side effects [**4**].

All couples who are unable to conceive after 12 months of regular, unprotected intercourse have indication for diagnostic procedures. Diagnostic approach will start with history taking and clinical examination and will continue with lab tests and possible needed diagnostic procedures in both men and women.

Male factors (as main cause or with female factor) are involving in 35% of all causes of infertility. Male factors are divided to 3 main causes: sperm production disorders, sperm functional disorders and vas deferens obstruction.

Sperm analysis (spermogram) is an essential important diagnostic study in male infertility diagnostic approach and usually is abnormal in infertile men. Unfortunately, infertility in most of men is idiopathic that shows lack of our knowledge from different mechanism of testis functions.

Environmental factors like heat, smoking, radiation, heavy metals and others can effect on spermatogenesis. Febrile diseases can cause considerable but reversible decrease in sperm count. Based on some theories heat generator environmental sources like jobs who need long time sitting (like driving) can cause infertility, although yet these have not been proved by clinical trials.

Cigarette has about 4000 chemical components like carbon monoxide, nitrogen oxide, hydrocarbons, tar, hydrogen cyanide, nicotine, etc. About 40 type on these components have are cancerogens [**5**]. 

Effect of cigarette on sperm function has been detected carefully but the mechanism has not been understood completely [**6**]. In many studies, the relation between smoking and semen analysis parameters changes (like morphology, motility and concentration) but there are some incoherencies about being a definitive etiologic cause of infertility or the relation between severity of smoking and infertility. Smoking, of course can increase inflammatory agents and effect on sperm genome and gonads and failure to sperm-ovum fecundation and decreased fertility [**7**].

Sperm analysis is an easy cheap lab test and always is a first diagnostic test to approach to infertile couples. Male infertility is usually concomitant with abnormal sperm analysis [**8**]. The abnormal test needs more detailed diagnostic approaches.

## Materials and methods

This cross sectional study was done on men from couples who were referred to Yazd Research and Clinical Center for Infertility during September 2009-october 2010. Cause of infertility was not male factors and spermograms of all cases were within normal ranges. Patients with history of varicocele, orchitis, testis trauma with hematoma, spinal injury, hernia repair, criptorchidsm, alcohol abuse (more than 4 glasses per week) and patients with positive history of using drugs like cimetidine, anti hypertensive, anti depressants, anti psychotics and chemotherapy, drugs abuse like morphine, cocaine, heroin and marry Juana were excluded from the study.

For sampling, the patient must avoid sex about 2-3 days before sampling. Longer or shorter intervals can effect on sperm analysis results and cause misinterpretation. Sampling should be by masturbation and direct pouring semen into a clean container directly. Some specific condoms are available for this purpose too, but ordinary condoms who are used for protection are banned because have spermicidal agents [**9**].

Sample can be analyzed in room or body temperature, but microscopic studies must be done in body temperature because room temperature changes can cause misinterpretation. Usually immediately after ejaculation sperm will be agglutinated; and 15-30 minutes is needed to become watery again. Being agglutinated for more than one hour is abnormal and needs a specific treatment. If there were no spermatozoa, liquid must be centrifuged and studied by microscope [**10**]. Reference values in sperm analysis [**11**] are indicated in **Table 1**.

**Table 1:** Reference values in sperm analysis are indicated

Parameter	Lower reference limit
Semen volume (ml)	1.5 (1.4-1.7)
Total sperm number (106 per ejaculate)	39 (33-46)
Total sperm number (106 per ejaculate)	15 (12-16)
Progressive motility (PR, %)	32 (31-34)
Sperm morphology (normal forms, %)	4 (3.0-4.0)
Vitality (live spermatozoa, %)	58 (55-63)
pH	≥ 7.2

Patients were allocated to smoker and non-smoker groups. Patients with history of smoking of more than 10 cigarettes per day, for more than 3 months were included in smoker group. 

Data from semen analysis including concentration, morphology and motility were registered and analyzed under ANOVA test by SPSS software. 

## Results

Results are shown in **Table 2** and **Table 3**.

**Table 2:** Mean values for concentration, motility and morphology in smoker and non-smoker groups and related P values.

Group	Number	Mean Concentration Million/ml	Standard deviation	P
smoker	53	86.7	41.06	
Non smoker	98	101.3	58.5	0.108
total	151	96.2	53.4	
Group	Number	Men Morphology (%)	Standard deviation	P
smoker	53	47.4	15.6	
Non smoker	98	51.08	13.4	0.138
total	151	49.8	14.3	
Group	Number	Mean Motility (%)	Standard deviation	P
smoker	53	72.3	7.14	
Non smoker	98	74.3	6.43	0.082
total	151	73.6	6.74	

**Table 3:** Relation between Packed/Years with morphology, Motility and concentration

Motility	Morphology	Concentration		
-0.190	-0.147	-0.241	Pearson correlation (r)	
0.174	0.297	-0.147	P-value	
53	53	53	number	

Totally, 151 patients were included in the study (98 as non-smokers and 53 as smokers). Mean age of smoker and non-smoker groups were 34±5.8 and 32.4±5.5 respectively. Mean values of concentration among smokers and non-smokers were 86.7±41.06 million per ml and 101.3±58.5 respectively (P=0.108). Mean packs/years for smoker groups was 6.2±6.08.

 Mean values for morphology were 47.4±15.6 and 51.08±13.4 among smokers and non-smokers respectively (P=0.138).

 Motility mean values were 72.3±7.14 and 74.3±6.43 in smokers and non-smokers (P=0.082).

 There were inverse correlation between pack/years and morphology, motility and concentration but P-Values were not significant.

## Discussion

Infertility involves about 10-15% of the couples of the world [**1**]. Male factors are 35% of all infertility causes. Smoking is an environmental factor that effects on semen. 

In Taszarek et al. study in Egypt, on 27 infertile smokers and 75 infertile non-smokers’ men, morphology and concentration were not statistically different; but motility was lower in smokers significantly [**10**]. These results were repeated in Hassan et al. study in Turkey [**12**]. In our study, there was no significant difference between the two groups according to morphology, concentration and motility.

In Pasqualotto FF et al. study in Brazil, 889 smoker men divided into four groups according to numbers of cigarettes that they were using. Patients were divided to non-smokers, mild smokers (<10 cigarettes per day), Moderate smokers (11-20 cigarettes per day) and heavy smokers (>20 cigarettes per day) groups. There were no significant difference between groups according to concentration, morphology and motility. This study shows that even the cigarettes that people use have no influence on sperm parameters [**13**]. These results and also the results of Sandrine et al. study in Ireland [**14**] are concomitant with our study. In our analysis, there was an inverse relation between numbers of cigarettes and sperm parameters, but they were not significant in any parameter. In addition, our results are similar to Hassan et al. study [**12**] that did not show any relationship between the number of cigarettes used and sperm parameters.

In 1993, Moskova et al. did a study in Bulgaria that dealt with the comparison of sperm parameters on 169 smoker and non-smoker men; they concluded that motility was decreased in smokers significantly but morphology was increased in smokers and concentration was not different between groups [**15**]. Results of this study were similar to our study according to concentration but not in morphology and motility.

As seen, there are conflicting results about the effect of smoking on sperm parameters. Of course, the distinctive specification of our study and also Hasan et al. [**12**] is our sampling, because we allocated people who had not proved to be infertile; then infertile men whose abnormal sperm analysis could affect the results were excluded. Also Pasqualotto FF et al. [**13**] had a larger sample size that could make their study be more valid. Moskova et al. [**15**] did not mention their allocation method explicitly and it is possible that they have included some people with infertility, who could have affected their results. Taszarek-H G et al. [**10**] mentioned that 27 infertile men who smoked cigarettes, 79 infertile men who were nonsmokers and 82 healthy nonsmoking donors were evaluated in his study, but the cause of infertility in smoker infertile men was not explained. This can be an important bias while possibly the cause of sperm analysis abnormalities in such patients was not due to smoking but also due to another cause of infertility.

## Conclusion

Semen parameters (Morphology, Motility and concentration) were lower in smokers but there were no significant differences between groups. However, it could be useful to compare the fertilization success (in couples with female factor causes) to detect if this mild non-significant differences can affect the success rate or not. Moreover, the detection of the effect of long time smoking on fertility could be indicated by choosing a homogenous sample size of people with heavy smoking higher for more years. This will omit the bias of young heavy smokers on results.

**Conflict of interest**

This study was under financial support of faculty of medicine, Shahid Sadoughi University of Medical Sciences, Yazd, Iran as part of Mr. **Naeimeh Naserzadeh** dissertation to be graduated as General Practitioner. Other authors declare no conflict of interest.
